# microRNA 146a ameliorates retinal damage in experimental autoimmune uveitis

**DOI:** 10.3389/fopht.2023.1130202

**Published:** 2023-03-24

**Authors:** Sindhu Saraswathy, Narsing A. Rao

**Affiliations:** ^1^Department of Ophthalmology, Doheny Eye Institute, Los Angeles, CA, United States; ^2^Department of Opthalmology, USC-Roski Eye Institute, University of Southern California, Los Angeles, CA, United States

**Keywords:** uveitis, experimental autoimmune uveitis, αA crystallin, microRNA 146a, mitochondrial oxidative stress

## Abstract

**Introduction:**

Uveitis and related intraocular inflammations are a major cause of blindness due to retinal damage caused by degeneration and loss of the photoreceptor cells. In mouse experimental autoimmune uveitis (EAU) previously we have shown mitochondrial oxidative stress with marked upregulation of αA crystallin in the inner segments of the photoreceptors. Furthermore, αA crystallin treatment prevented photoreceptor mitochondrial oxidative stress by suppressing innate and adaptive immunity in EAU.

**Methods:**

Since these immune processes are modulated by microRNAs, in this study we investigated (a) modulation of microRNAs during development of EAU by αA crystallin administration and (b) microRNA therapeutic intervention.

**Results:**

Few microRNAs were significantly upregulated in EAU mice with intravenous injection of αA crystallin and among these, computational bioinformatic analysis revealed that the upregulated microRNA 146a targets the innate and adaptive immune responses. In EAU, intravenous as well as intravitreal administration of this microRNA prevented inflammatory cell infiltration in uvea and retina and preserved photoreceptor cells.

**Discussion:**

This protective function suggests that microRNA146a can be a novel therapeutic agent in preventing retinal damage in uveitis.

## 1 Introduction

Uveitis, an intraocular inflammation which primarily involves the retina and the uvea, is a major cause of blindness due to retinal photoreceptor degeneration from mitochondrial oxidative stress (1, 2). The mitochondrial oxidative stress and damage primarily takes place in the retinal photoreceptors (3, 4). Experimental Autoimmune Uveitis (EAU) is well- established animal model of uveitis. This model has been used to decipher and study the efficacy of novel anti- inflammatory therapeutics and their mode of action to investigate the molecular mechanisms of the disease progression (5). We have earlier reported a novel finding of selective αA crystallin upregulation in the photoreceptors during the early phase of EAU (6). This upregulation occurred at the sites of the mitochondrial oxidative stress in the retina, mainly in the photoreceptor cells. Moreover, systemic administration of αA during the EAU development resulted in protection of the retina and amelioration of the inflammatory process by down regulating the TLRs, Th1- and Th17 cytokines required for induction of oxidative stress and perpetuation of the intraocular inflammation (7). However, the molecular mechanism of such αA crystallin mediated suppression of proinflammatory cytokines and TLRs was unclear. Recent studies in inherited retinal degeneration mouse model also reported a similar finding indicating that loss of α crystallins resulted in increased intraretinal inflammation, activation of apoptosis, necroptosis and photoreceptor death suggesting its important role in regulating photoreceptor survival (8). Crystallins, the major structural proteins of the eye lens, consist of three distinct families: α, β and γ (9). The α-crystallins constitute two similar crystallins with about 60 percent homology: αA and αB, and these are the principal members of the small heat shock proteins (sHsps) family of molecular chaperones which are known to be expressed in multiple tissues, including retina, brain, heart, kidney, spinal cord, and lungs (10–15). Although αA- and αB-crystallin have related amino acid sequences with similar structural properties, they vary significantly in their tissue specificity and phosphorylation sites and have different functions (16, 17). Since α -crystallins are induced by a temperature upshift in many organisms, they are often referred to as small heat shock proteins (sHsps) or, more accurately, alpha-Hsps. sHSPs are found to modulate microRNA expression and through such modulatory effect they could suppress autoimmune process (18). MicroRNAs (miRNAs) are a class of noncoding RNAs 18-24 nucleotides long and suppress the expression of protein-coding genes at the post transcriptional level by directing translational repression, or by destabilizing mRNA or by a combination of the two (19–22). They are transcribed in the nucleus through transcription factor NFκB and are exported into the cytoplasm where they control gene expression at the post transcriptional level by binding to target messenger RNAs (23). As binding sites of these microRNAs are not fully complementary; each miRNA may target several different mRNA transcripts. As a result, modulation of individual miRNAs can change the expression of hundreds of genes. These RNAs are known to play key roles in a wide variety of biological processes, including cell differentiation and growth, development, metabolism, cell signaling, apoptosis and disease processes linked to cancer and inflammation (20, 24–26). MicroRNAs are also promising candidates for new, targeted therapeutic approaches and as biomarkers of diseases like cancers, heart disease and chronic inflammatory diseases (23). miRNA-based therapies involve modulation of pathogenetic pathways by antagonists and mimics. MicroRNA biogenesis occurs through activation of NFκB, and studies have shown that the small heat shock protein Hsp27, a close member of the αA-crystallin family, regulates NFκB activity through degradation of IκB leading to upregulation of miRNA biogenesis (18). We postulated that the beneficial effect of αA administration in amelioration of uveitis could be from the αA modulation of NFκB transcription factor. Although uveitis is caused by various infectious agents and develops in association with autoimmune systemic disorders, the later uveitis entities are commonly encountered in clinical practice and they are usually treated with non-specific immune modulators including corticosteroids, cytotoxic agents, and biologicals such as anti-TNFα (1, 27, 28). However, such therapeutic interventions are associated with significant systemic complications such as systemic immune suppression, infections, malignancies and ocular complications, glaucoma, and cataract (29). Interestingly, the above therapeutic agents were delivered locally in the uveitis eyes by intravitreal injections and such treatments were effective, however such local delivery was also associated with ocular complications, cataract development and glaucoma (30). In contrast microRNAs by virtue of their function in suppressing the proinflammatory cytokines genes (21) could offer advantages in treatment of uveitis by local delivery because of low toxicity, absence of degradation and efficient delivery to the retina and uvea due to their low molecular size. In this study of EAU, we carried out modulation of microRNA expression profile followed by bioinformatics analysis combined with validated microRNA target data to detect specifically upregulated miRNAs which are known to suppress inflammatory cytokines. Moreover, we determined the abrogation of EAU by systemic and local administration of the miRNAs.

## 2 Material and methods

This study was carried out in strict accordance with the recommendations in the Guide for the Care and Use of Laboratory Animals of the Association of Research in Vision and Ophthalmology. The protocol was approved by the Committee of the Ethics of Animal Experiments of the University of Southern California (Protocol Number: 11218). All surgery was performed under ketamine and xylazine anesthesia, and all efforts were made to minimize suffering of animals.

### 2.1 MicroRNA expression profile in EAU animals treated with αA crystallin

EAU was induced in two groups of 12 B10RIII mice as previously described by immunizing with 25 μg of Interphotoreceptor retinoid-binding protein (IRBP) peptide 161–180 (SGIPYIISYLHPGNTILHVD) in phosphate buffered saline (PBS) emulsified 1:1 vol/vol in complete Freund’s adjuvant that had been supplemented with *Mycobacterium tuberculosis* to 2.5 mg/ml (6, 31). 12 mice without EAU served as controls. Beginning on day12 post immunization (pi) with IRBP one group of mice was intravenously injected with αA crystallin (10µg in normal saline) on alternate days. Presence of endotoxin in the αA crystallin recombinant protein was determined using the Limulus amebocyte lysate (LAL) assay (Thermo scientific Pierce LAL chromogenic endotoxin quantitation kit, Rockford, IL) (32, 33). Samples were measured on a microplate absorbance reader at 405nm. A standard curve was created using the E. coli endotoxin standard included with each kit to calculate endotoxin levels. The second group of EAU animals similarly received normal saline devoid of the crystallin. All 36 mice were killed on day 21 and the retinas were subjected to mouse whole genome microRNA PCR array (Qiagen). The experiments were performed in triplicate. MicroRNA expression profiles were then subjected to statistical analysis using T-test and the results showing significant fold increase or decrease with P<0.05 were selected. The altered levels of microRNA, 50% above or below normal were considered significant as per previous reports (34).

### 2.2 Computational bioinformatics analysis to study the target genes of microRNAs

The significantly altered microRNA profile results from the experiment 1 was subjected to bioinformatics calculation limitation and P-values (P<0.05) stringency tests. A target search for the modulated microRNAs was then carried out with miRWalk software, and other validated databases miRBase, and TarBase (35, 36). To prioritize the list of targets and determine the cellular processes that are most significantly affected by the microRNAs in EAU, a pathway enrichment tool analysis was performed. The pathway analysis tool grouped the microRNAs according to the pathways in which they were involved (37).

### 2.3 Interaction of αA crystallin with NFκB in αA knock out EAU animals

EAU was induced in 2 groups of αA knock out (KO) mice (129SvEv mice provided by Dr. Eric Wawrousk) twelve animals in each group by immunizing with 25µg of IRBP. One group of 12 KO mice without EAU served as controls. One group of αA KO mice with EAU were treated with 10µg of αA Crystallin recombinant protein (free of LPS contamination as shown in the previous experiment) every 48 hours from day 12 through day 20. Another group of EAU mice were similarly treated with a 10µg of non-relevant protein (αB crystallin which was found to have no effect in EAU) as we have earlier reported (6, 7). The mice were sacrificed on day 21. The eyes were enucleated, and retinas were subjected to western blot analysis to determine levels of phosphorylated iκB-α and activated NFκB (p65) using specific antibodies (sc-8404, anti-mouse p-IκB-α (1:1000), sc-136548 p-NFκB p65 (1:1000); Santa Cruz biotechnology). For this equal amount of the retinal proteins were subjected to electrophoresis on 12% SDS-polyacrylamide gels and transferred to nitrocellulose membrane using semi-dry transfer apparatus (Millipore, USA). The membrane was blocked with 10% milk protein and sequentially incubated with appropriate primary antibodies for phosphorylated iκB-α and activated NFkBp65 and HRP-conjugated secondary antibodies and visualized using Amersham-Pharmacia ECL detection kit. Glyceraldehyde 3-phosphate dehydrogenase (GAPDH) was used as the normalizing protein.

### 2.4 Determine the severity of EAU in animals with microRNA 146a knock out and wild type mice

Twelve microRNA 146a KO mice (genetic background:C57BL/6, Jackson Lab) were used for this experiment. At 6-8 weeks of age, homozygotes of this strain of mice do not display a visible autoimmune or inflammatory phenotype. 12 microRNA 146a KO mice and 12 control WT mice (C57BL/6) were immunized with 25ug of IRBP as previously described (7). The Mice were sacrificed on day 21 and the enucleated eyes were fixed in 4% formalin, embedded in paraffin, and sectioned for Hematoxylin-Eosin (H&E) staining. Eye sections cut through pupillary-optic nerve planes were scored in a masked fashion. Severity of EAU was determined from the H&E staining and graded on a scale of 0 to 4 in half-point increments using the criteria, based on lesion type, number, and size as described earlier (38–40). A group of 12 non-immunized KO and WT mice were sacrificed, and the enucleated eyes were similarly examined. For immunohistochemistry the eyes were fixed in Tissue-Tek OCT compound and 10 -micrometer cryosections of the retina were fixed in 4% paraformaldehyde for 20 min and subsequently stained using anti-IRBP (sc -390218, 1:100, Santa Cruz Biotechnology, CA). The immunohistochemical sections were visualized using confocal microscope. (Carl Zeiss, Oberkochen, Germany). Isotype controls and PBS replaced primary antibody were used as negative controls. The experiments were performed in triplicate. The analysis for quantification of the average mean fluorescence intensity of IRBP staining was done using QuPath analysis software version 0.2.3. QuPath is a user-friendly bioimage analysis software, and its algorithms can be used to analyze and quantitate specific proteins in specific cells of interest (41, 42).

### 2.5 Amelioration of EAU by systemic administration of microRNA 146a

In 72 B10RIII mice, EAU was induced with IRBP as described earlier. Thirty-six mice without EAU served as controls. Starting on day 12 post immunization (p.i) with IRBP, one group of 36 EAU mice received an intravenous tail vein injection of 1µl of 10µmol/l microRNA 146a or microRNA155 (Dharmacon) every other day. Another group of 36 EAU mice received injections of 1µl of 10µmol/l of a scrambled microRNA sequence. On day 21 pi, all animals were sacrificed.

The eyes from 6 mice from each group were enucleated, fixed in OCT and retinal cryosections were used for immunohistochemistry using anti-IRBP antibody as described before. The paraffin retinal sections from 6 mice were processed to detect for apoptotic cells employing TUNEL assay (Roche Diagnostics, IN). DNAse1 treated tissues was used as the positive control and no enzyme as the negative control. Staining was performed in triplicate. The retinal sections were also examined histologically by H & E staining for detecting the severity of EAU in the different groups of mice as described earlier.

Retinas from 6 mice from each experimental group were dissected out and processed to determine the Th1/Th2/Th17 cytokines by Multi-Analyte ELISArray kit (Qiagen) which analyses a panel of 12 cytokines (7). Total protein was extracted from the retinas from each group according to manufacture instructions and 40ug of protein was used for the assay to detect each cytokine. Experiment was done in triplicate.

T reg cell activation markers and TLR and signaling molecules were analyzed by qPCR assay. For this assay, retinas from 6 mice from each group were dissected out for RNA extraction (TriZol reagent, Invitrogen). The cDNAs were generated (Omniscript RT kit; Qiagen, Valencia, CA) and PCR was conducted with gene specific primers for IRAK1, FOXP3 and TRAF6 using the I-cycler (BioRad). Glyceraldehyde-3-phosphate dehydrogenase (GAPDH) was used as the normalizing gene as it did not show any significant changes in our experimental protocols. (**Table 1**) (43, 44) The PCR reactions for each gene in each experiment were performed in triplicate on each cDNA template, along with triplicate reactions for the housekeeping gene GAPDH. The threshold cycle (Ct) difference between the experimental (mir146a treated) and control groups (Scrambled RNA treated), for each gene in each tissue, was calculated and normalized to GAPDH, and the increase (*x*-fold) in mRNA expression was determined by the 2-ΔΔct method. Statistical analysis of ΔΔCt was performed with a student’s *t*-test for three independent samples, with significance set as *P*<0.05 and compared between the different experimental groups.

**Table 1:** Primer sequences of TLR signaling molecules IRAK1 and TRAF6 and Treg marker FOXP3. The normalizing gene used was GAPDH.

Genes	Primer Sequences	
Foxp3	GGACAGACCACACTTCATGCA	GCTGATCATGGCTGGGTTGT
TRAF6	ATTTCATTGTCAACTGGGCA	TGAGTGTCCCATCTGCTTGA
IRAK1	GAGACCCTTGCTGGTCAGAG	GCTACACCCACCCACAGAGT
GAPDH	GGTGAAGGTCGGTGTGAACG	TGTAGACCATGTAGTTGAGGTCA

### 2.6 Amelioration of EAU from local (intravitreal) administration of microRNA 146a

Twelve B10RIII mice were immunized with IRBP. A group of six EAU mice received intravitreally delivered microRNA 146a (1µl of 5µmol/l) labeled with DY547 (Dharmacon) and another group of six mice were treated similarly with 1µl of 5µmol/l scrambled microRNA sequences labeled with DY547 on day 12 p.i. Mice were killed on day13 and14 p.i, and the retinal cryosections were visualized by confocal microscopy (Carl Zeiss, Oberkochen, Germany). PBS replaced primary antibody were used as the negative controls. The experiments were performed in triplicate. An additional twelve B10RIII mice were immunized with 25µg of IRBP as described earlier. One group of six EAU mice were intravitreally injected with mimic of microRNA 146a (1µl of 5µmol/l) (Dharmacon) every 48 hours from day 12 p.i. A second group were similarly treated (1µl of 5µmol/l) with scrambled microRNA sequence. Mice were killed on day 21 and the eyes were fixed in formalin. The paraffin sections were stained with H&E. The experiments were performed in triplicate.

### 2.7 TLRs and their signaling molecules, TH1, TH2 and TH17 cytokine profile in EAU animals treated with intravitreal delivery of microRNA

Twelve B10RIII mice were immunized with IRBP and a group of six EAU mice were injected intravitreally with microRNA 146a (1µl of 5µmol/l) and another group of six mice were similarly treated with (1µl of 5µmol/l) scrambled microRNA sequence every 48 hours from day 12p.i. A group of 6 mice without EAU served as controls. Mice were sacrificed on day 18 and the retinas and spleens were dissected out and subjected to qPCR array to detect TLRs and their signaling molecules, TH1/Th2/Th17 cytokines at mRNA level. RNA extraction was performed by the TriZol method (Invitrogen, Carlsbad, CA). RNA was quantified and checked for purity and integrity by microanalysis in an Agilent Bioanalyzer (Agilent Technologies, Santa Clara, CA). These RNAs were used to prepare cDNAs, which was used for PCR array analyses (SABiosciences, MD). All the three experimental groups were compared to each other. The experiments were run in triplicate.

## 3 Results

### 3.1 αA crystallin up regulates microRNA 146a in EAU animals

The LAL assay of recombinant αA and αB proteins showed no traces of endotoxin in these proteins. Compared to non-EAU controls, the EAU mice on post immunization day 21 the microRNA PCR array showed significant (P<0.05) downregulation of 15 microRNAs including microRNA 146a (-1.6) and microRNA 155 (-1.5) (**Table 2**). However, intravenous injection of αA crystallin upregulated these microRNAs.

**Table 2:** Modulating microRNA expression in EAU with and without αA treatment.

MicroRNAs	Fold change in EAU mice without treatment compared to non-EAU mice	Fold change in EAU mice with αA treatment compared to non-EAU mice
microRNA 146a	**-1.6**	**2.85**
microRNA 155	**-1.5**	**2.93**
microRNA 466g	**-1.5**	**2.24**
microRNA 200a	**-1.7**	**2.57**
microRNA 295	**-3.23**	**2.3**
microRNA 295	**-2.16**	**2.1**
microRNA 291b-3p	**-1.8**	**2.75**
microRNA 466t-5p	**-1.7**	**2.49**
microRNA 801	**-2.8**	**2.27**
microRNA 615	**-2.03**	**2.24**
microRNA 542-3p	**-2.13**	**2.13**
microRNA 199b	**-2.44**	**2.5**
microRNA 208b	**-2.09**	**2.27**
microRNA 429	**-2.07**	**2.54**
microRNA 1	**-2.7**	**2.17**

Increased expression of microRNA146a and other microRNAs in the retina of EAU mice after systemic administration of αA crystallin. EAU was induced in B10RIII mice and the retinas on day 21 were subjected to mouse whole genome microRNA PCR array. Altered levels of microRNA, 50% or above were considered significant and statistical analysis was performed using t-test and P<0.05 was considered significant. Bold negative values (left column)represent fold change -downregulation and Bold values in right column represent fold change -upregulation.

Among these upregulated microRNAs 146a and 155 showed 2.85- and 2.93-fold increase compared to non-EAU control animals. The levels of increases in the other microRNAs are shown in **Table 2**

### 3.2 Bioinformatics reveals microRNA 146a targets genes of TLR signals

Of the 15 microRNAs which were altered during EAU, were subjected to the computational bioinformatics analysis using validated databases miRWalk, miRBase and TarBase and a pathway enrichment analysis tool to determine the microRNAs known to be involved in inflammation pathways. Results showed that the two upregulated microRNAs, microRNA146a and microRNA155 played a role in the regulation of inflammation; microRNA 146a targets several genes of the innate and adaptive immunity whereas the gene targets of microRNA155 are limited. Computational bioinformatics analysis and pathway enrichment tool software revealed several validated and potential targets for microRNA 146a. The targets include TLR4, MyD88, IRAK1(IL-1 receptor associated kinase) and TRAF6 (TNF receptor- associated factor 6) related to innate immune response and TNF-α, IL-12, IL-17, and IFN-γ, related to adaptive immunity. The validated targets of microRNA155 were MyD88, IFN γ and TNF α.

### 3.3 αA crystallin activates NFκB in EAU animals

**Figure 1:**
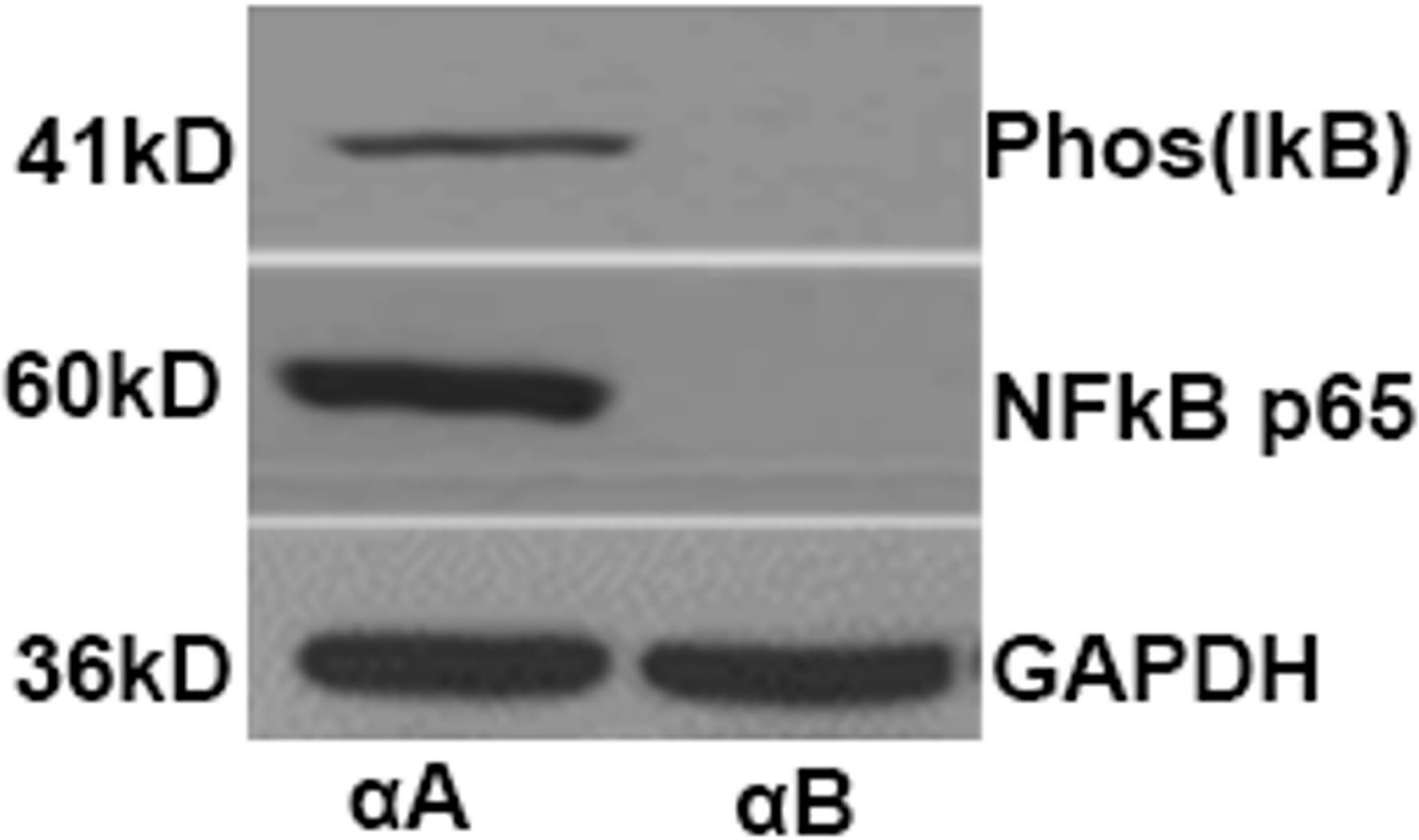
αA-Crystallin enhances phosphorylation and subsequent degradation of IκBα as well as activation of NFκB in EAU. Western blots showing the levels of phospho-IκBα and activated NFκBp65 from retinal protein lysates of mice treated with αA crystallin. Such changes are absent in EAU animals treated with αB crystallin.

Phosphorylated iκB which is formed by IκB kinase complex enzymes leading to its ubiquitination and degradation was present in the retinas of αA crystallin treated mice (**Figure 1**). This is known to lead to the activation of NFκB and subsequent translocation of the molecule to the nucleus. In the nucleus, NFκB binds with various genes and thus activates their transcription including microRNA146a (45–48). Phosphorylated iκB was absent in the EAU mice with αB crystallin treatment. Similarly increased levels of activated NFkB p65 was detected in the αA crystallin treatment groups whereas the activated NFkB was absent in the group of mice treated with αB crystallin (**Figure 1**).

### 3.4 Severe EAU in microRNA146a KO compared to WT mice

**Figure 2:**
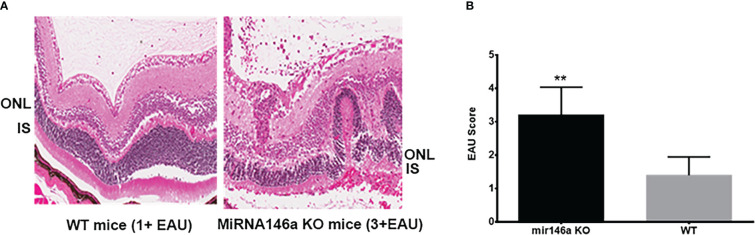
Severe inflammation in the microRNA146a KO mice compared to wildtype EAU mice. C57BL/6 mice develop milder EAU. H & E of representative histology section shows severe inflammation in the microRNA 146a KO mice 3 to 4+ associated with extensive retinal damage compared to the WT animals with 1 to 2+ EAU with relatively mild to moderate retinal damage **(A, B)** Bar graph represents EAU Score **P<0.001.

**Figure 3:**
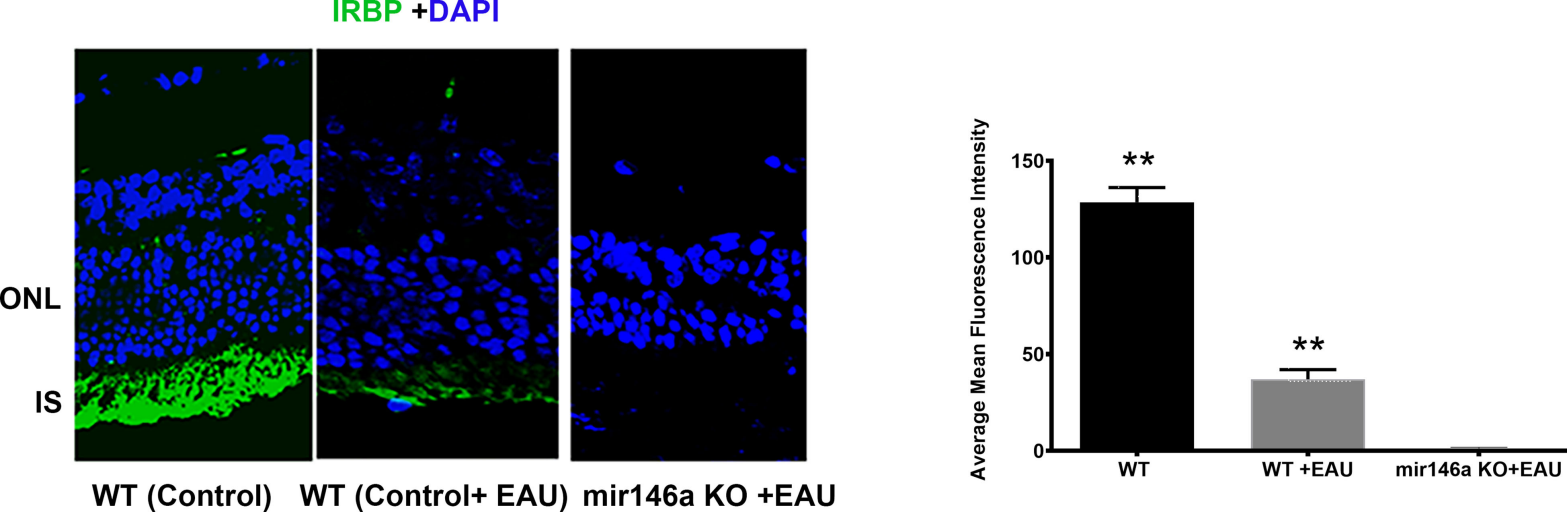
Immunohistochemistry for IRBP reveals total loss of IRBP in the KO mice compared to WT mice with EAU. Control wildtype (C57BL/6) without EAU; (WT) C57BL/6 with EAU; C57BL/6 microRNA146a KO with EAU. Note some preservation of photoreceptors in WT with EAU when compared to KO mice, which showed total loss of green staining. **P<0.001(WT control compared to WT+EAU and mir146aKO+EAU; WT EAU compared to mir146aKO+EAU).

The microRNA 146a KO animals with EAU showed severe inflammation of 3 to 4+ associated with extensive retinal damage compared to the WT animals with 1 to 2+ EAU score with relatively mild to moderate retinal damage **(** **Figure 2**, ** (p<0.001). Inflammation in all the microRNA146a KO mice with EAU was more prominent than the WT mice and interestingly the retinal photoreceptor damage was more pronounced in KO animals compared to WT mice **(** **Figure 2****).** No IRBP staining could be detected in the microRNA146a KO mice with EAU whereas a minimal staining was present in the WT mice with EAU compared to non-EAU animals **(** **Figure 3**, ** p<0.001)).

### 3.5 microRNA 146a systemic administration abrogates EAU

**Figure 4:**
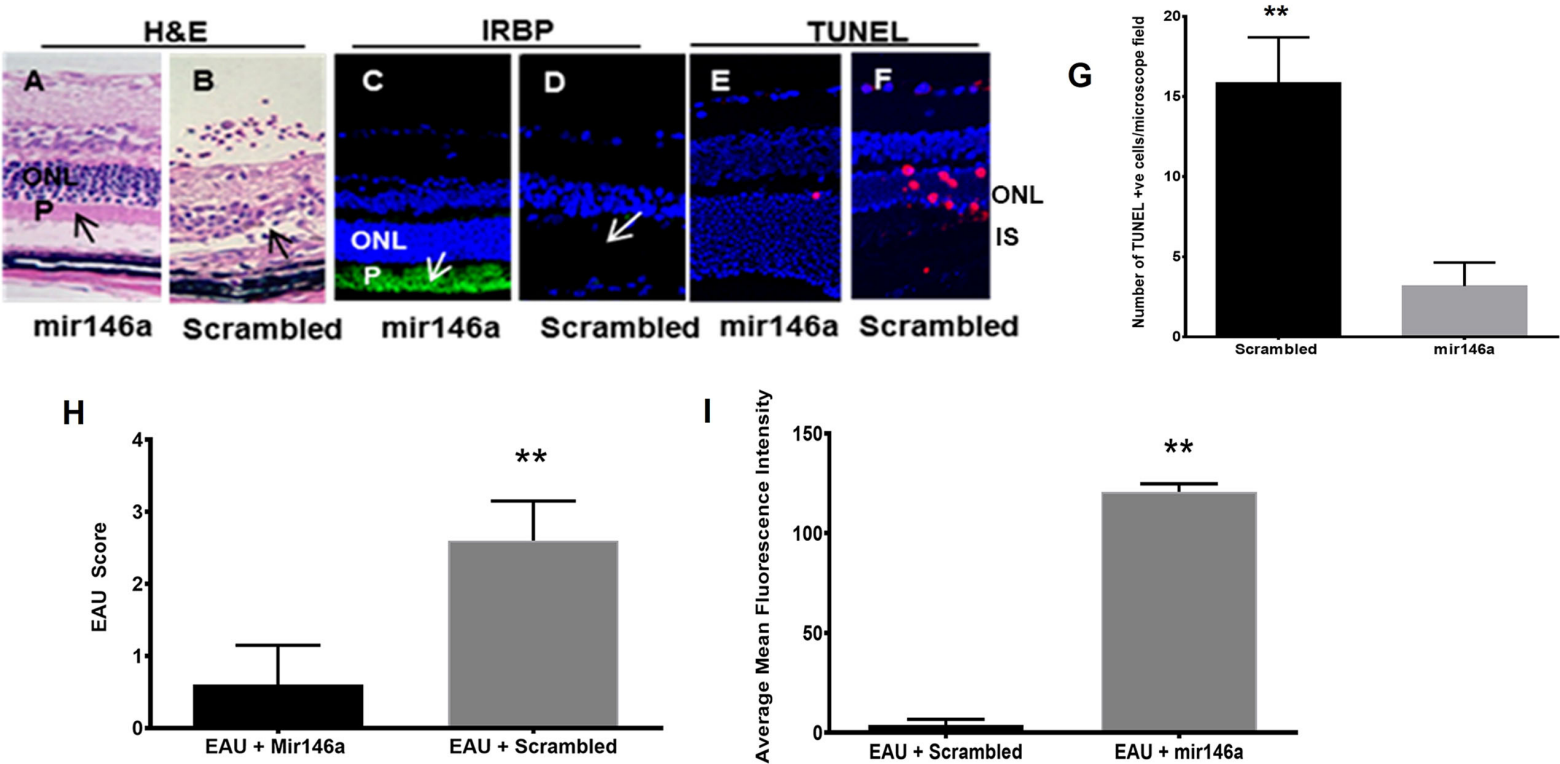
Abrogation of EAU by microRNA146a. EAU was induced in B10RIII mice. A group of mice were treated with microRNA146a and another group with scrambled microRNA sequences. The retinal paraffin embedded sections from these mice were stained for H&E staining. Another group of retinal cryosections from these group of mice were immunostained with IRBP antibody. Retinal sections were also subjected to TUNEL staining using a kit according to manufactures instructions. EAU mice treated with microRNA146a showed abrogation of EAU, retinal protection, and preservation of photoreceptors arrows; panels **(A, C)**. Such protection is absent in mice treated with scrambled microRNA sequences (arrows: panels **(B, D, H, I)** (**p<0.001)). **(C, D, I)**, represent immunohistochemistry for IRBP and **(E, F)** show TUNEL stain. **(E)** Mir146a and **(F)**; scrambled RNA treated. ONL: outer nuclear layer; P: photoreceptor layer. **(G)**. Bar graph showing significant TUNEL positive cells in Scrambled RNA treatment compared to mir146a treatment group. (**P<0.001).

**Figure 5:**
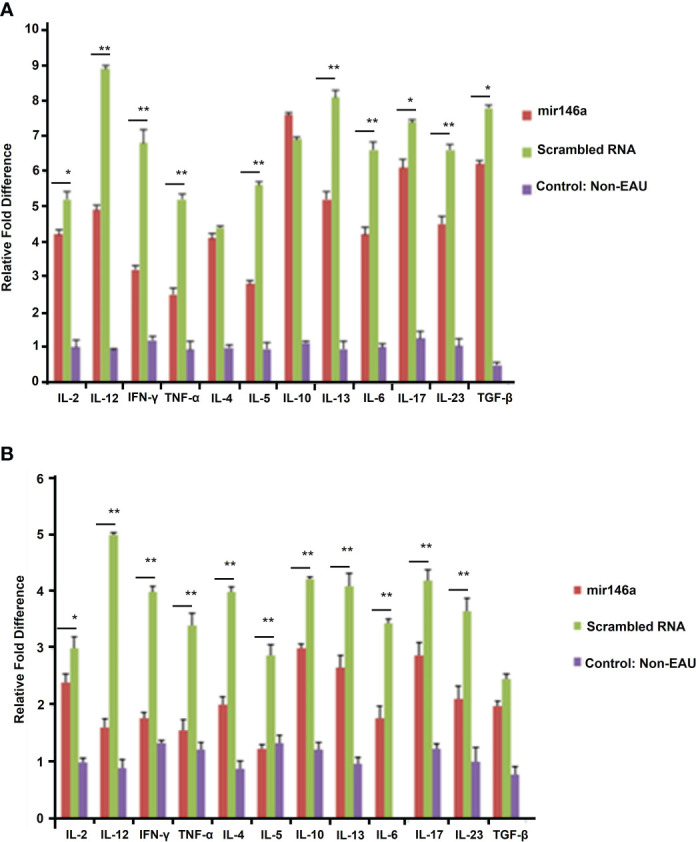
microRNA146a administration markedly lowers protein levels for various cytokines. Multi ELISArray shows that systemic microRNA146a treatment downregulates Th1/Th2/Th17 cytokines compared to scrambled microRNA sequences in retina **(A)** and spleen **(B)**. Control=non-EAU. ELISA assays show decreased levels of IL-2, IL-12, IFN-γ and TNF-α and Th17 cytokines IL-6, IL-17, IL-23, and TGF-β in EAU mice treated with microRNA146a. Error bars are standard deviation of the mean. * = P<0.05, ** = P<0.01.

EAU mice treated with microRNA 146a showed minimal or no inflammation and the retinas were well preserved with no damage. In contrast, the animals injected with the scrambled sequence revealed intraocular inflammation of 2+ to 3 EAU Score with retinal damage **(** **Figures 4A, B, H** (** p<0.01)**)**. Immunohistology of IRBP also showed preserved photoreceptors in the eyes of animals treated with microRNA 146a **(** **Figures 4C, D, I** (** p<0.001)**)** compared to animals treated with the scrambled microRNA sequence. TUNEL assay revealed the presence of occasional apoptotic cells in the retinas of EAU mice treated with MicroRNA 146a whereas there were numerous apoptotic cells in the mice injected with the scrambled sequence **(** **Figures 4E–G** ** p<0.001)). The protein levels of Th1/Th17 cytokines were significantly downregulated in the retina (**Figure 5A**) and spleen **(** **Figure 5B****)** of the microRNA 146a treated groups compared to the animals treated with scrambled RNA (*p<0.05, ** p<0.01)). PCR array revealed that IRAK1 and TIRAF6 were significantly downregulated in the retina as well as in the spleen in the EAU mice treated with microRNA 146a compared to the group of mice treated with scrambled RNA (*P<0.05, **P,0.01). In contrast the markers for activated T reg cells such as FOXP3 were upregulated in the spleen (P<0.05) after treatment with microRNA 146a (**Table 3**).

**Table 3:** Downregulation of TLR signaling molecules IRAK1 and TRAF6 after systemic injection of microRNA146a in EAU mice.

Genes	WT EAU treated with scrambled RNA (Retina)	WT EAU treated with mir146a (Retina)	WT EAU treated with scrambled RNA (spleen)	WT EAU Treated with mir146a (spleen)
**IRAK1**	6.06	2(P<0.05)	3.44	1.55 (P<0.05)
**TRAF6**	4.04	1.8 (P<0.05)	2.12	1.41 (P<0.05)
**FOXp3**	Not Done	Not done	1.3	2.2 (P<0.05)

EAU was induced in B10RIII mice, and the retinas and spleens were subjected to qPCR to detect the mRNA expression of TLR signaling molecules IRAK1 and TRAF6 as well as the T reg cell activation marker FOXP3. IRAK1, TRAF6 were significantly downregulated in the mice treated with intravitreal injection of microRNA146a compared to the scrambled microRNA sequences. FOXP3 was upregulated in the spleen after miRNA injection.

### 3.6 Local (intravitreal) delivery of microRNA 146a abrogates EAU

**Figure 6:**
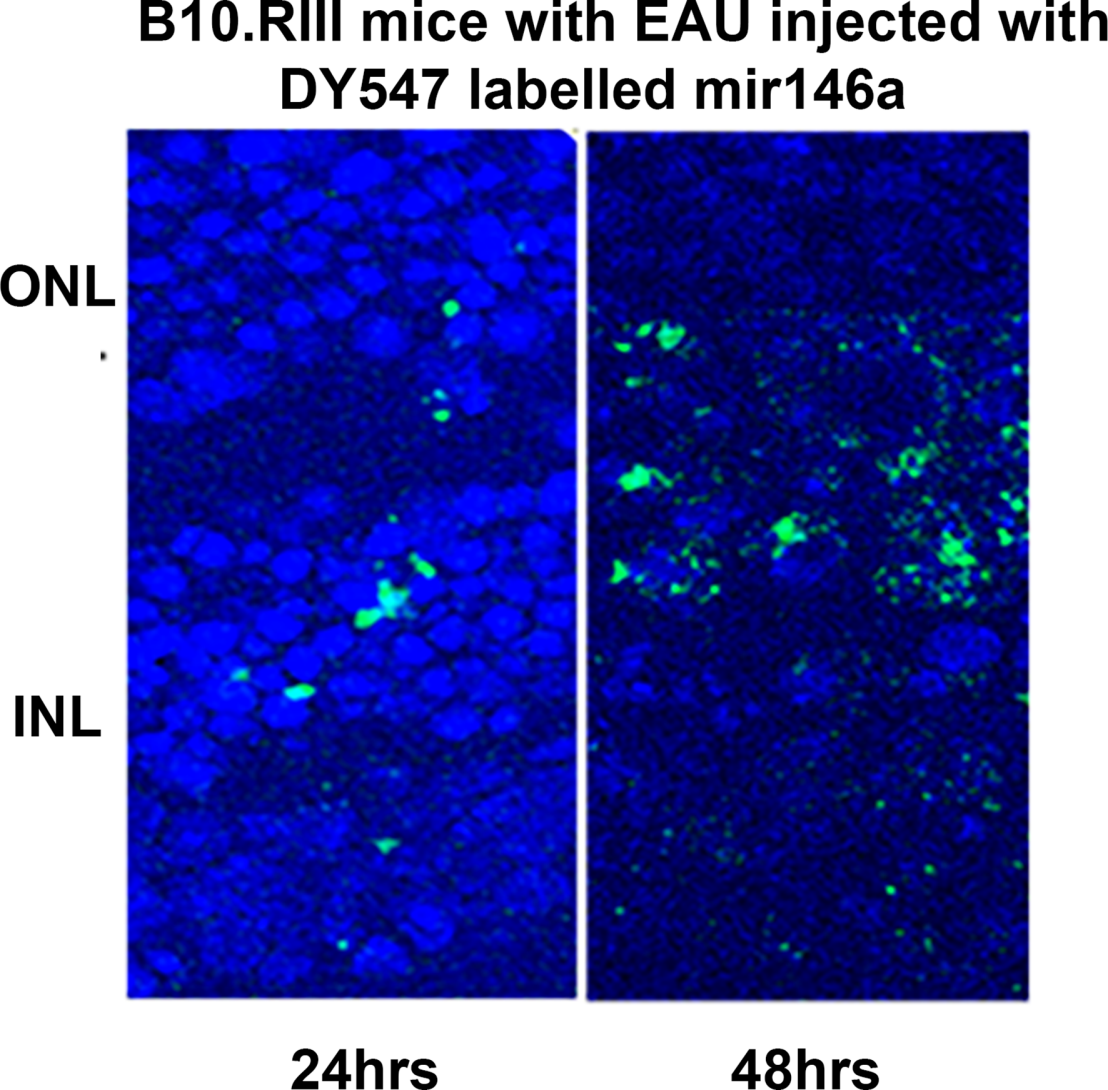
Localization of the labeled microRNA146a in the photoreceptor cells after intravitreal injection in EAU mice. DY547 labeled microRNA 146a was injected intravitreally in EAU mice to track the microRNA. The figure shows the labelled microRNA146a in the retina of the treated animals in the outer nuclear layers of the photoreceptors after 24 and 48 hrs.

**Figure 7:**
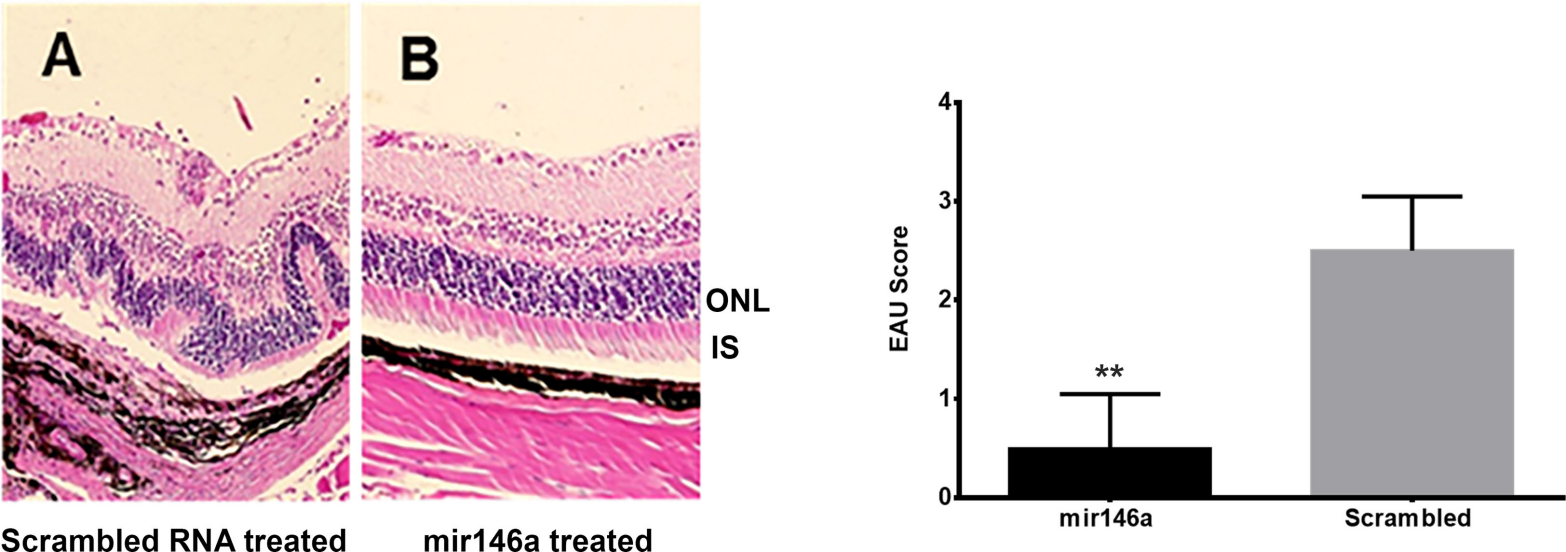
Abrogation of EAU by intravitreal delivery of microRNA146a. B10RIII mice was induced with EAU and from day 12 were treated with microRNA146a and scrambled microRNA sequences. The eyes from these mice on day 21 post immunization was subjected to H&E staining. **(A)** Local treatment with scrambled microRNA sequences. Note retinal damage in EAU animals treated with scrambled RNA. **(B)** Treatment with microRNA146a and abrogation of EAU. Bar graph represents EAU Score (**P<0.001).

The DY547 labeled microRNA 146a was localized in the outer and inner nuclear layers after 24 hrs and 48 hrs. **(** **Figure 6****).** Enhancement of this label was seen in the outer and inner nuclear layer of the retina. In the scrambled -treated mice, there was no label. The eyes treated with scrambled microRNA showed inflammatory cell infiltration in the retina and uvea with retinal damage typical of EAU, whereas the eyes treated with microRNA 146a had no inflammatory cell infiltration and the retina was well preserved**. (** **Figure 7**, **P<0.001**)**.

### 3.7 In EAU local delivery of microRNA 146a results in suppression of immune reactive molecules in the retina

**Figure 8:**
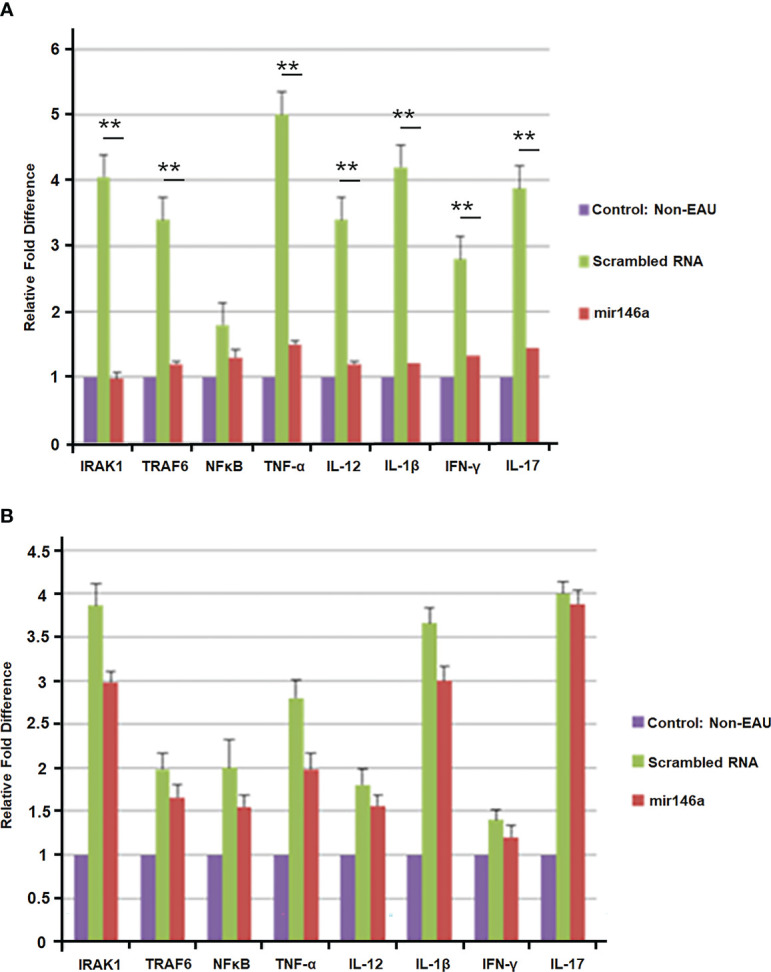
**(A)** Suppression of immune reactive molecules in the retina after intravitreal delivery of microRNA146a. B10RIII mice were induced with EAU and groups of mice were treated with intravitreal injection of microRNA146a and scrambled microRNA sequences from day 12 p.i. The retinas from these mice were dissected out on day 21 and subjected to qPCR array. The TLR signaling molecules and TH1/Th2/Th17 cytokines are downregulated in the retina of microRNA146a treatment mice. Statistical analysis was done comparing the Scrambled RNA versus microRNA146a treated groups. (*p<0.05, **p<0.01). **(B)** Absence of suppression of immune reactive molecules in the spleen after intravitreal delivery of microRNA146a. In the spleen there is no significant downregulation of these molecules at mRNA level in the microRNA146a treated group compared to scrambled RNA treated mice. Error bars represent standard deviations of the mean.

In the retina of the EAU mice treated with local delivery of microRNA 146a compared to those treated with scrambled RNA, there was suppression of IRAK1 and TRAF6. Similarly, Th1/Th2 and Th17 cytokines such as TNF-α, IL-12, IFN-γ and IL-17 mRNA were also significantly suppressed (**P<0.01). The levels of these mRNAs in the retina were similar in microRNA 146a-treated mice compared to non-EAU control animals **(** **Figure 8A****).** In contrast, in these treated animals, the spleen showed upregulation of the TLRs signaling molecules, IRAK1 and TRAF6 and the Th1/Th2/Th17 cytokines in both mirRNA146a as well as scrambled RNA treated group (**Figure 8B**).

## 4 Data analysis

Statistical analyses were performed by a student *t*-test using Prism (GraphPad Software, La Jolla, CA). A p value of < 0.05 was considered statistically significant.

## 5 Discussion

Development of experimental autoimmune uveitis requires activation of innate immune response leading to T cell mediated induction of organ specific autoimmune process directed at the retina. Recent studies on target prediction analyses have indicated that a significant number of innate immune response genes could be under the direct control of microRNAs (19, 49). Therefore we investigated the microRNA expression profile in the retina of the EAU mice treated with αA crystalline. Such treatment revealed upregulation of a few selective microRNAs in the retina. Moreover, these microRNAs were downregulated significantly in EAU retina without αA Crystallin treatment suggesting that αA Crystallin could upregulate select few microRNAs. (**Table 2**) However further studies with additional experiments are required to support the suggested role of αA crystallin in modulation of microRNAs.

Novel bioinformatics combined with validated microRNA targets of upregulated microRNAs from αA administration revealed that among these, microRNA 146a and 155 could play a significant role in inhibition/suppression in immune reactive genes resulting in the amelioration of the inflammatory process involved in uveitis (50–52). Computational bioinformatics analysis using validated microRNA targets showed that microRNA146a targets the mRNAs of key innate immune response genes of TLRs signaling molecules IRAK1 and TRAF6. These signaling molecules are crucial in initiation of robust innate immune response. This microRNA is known to negatively regulate important factors in the MyD88/NFkB signaling pathway, namely TRAF6 and IRAK1, thereby dampening the pro-inflammatory response mediated through MyD88/NFkB signaling (45, 51, 53–55). microRNA146a was also shown to reduce intraocular inflammation in Experimental Autoimmune Anterior Uveitis (EAAU) in rats through the inhibition of NFκB (56). Previous studies on TLRs signaling blocking experiments showed inhibition of EAU development implicating important role played by the innate immune response in induction of EAU (57). Blocking of various TLRs using TLR knockout mice did not prevent EAU however studies in MYD88 KO mice have shown that these mice were completely resistant to EAU as they fail to generate the proinflammatory and Th1 responses (58). However, inhibition of the downstream key signaling molecules of TLRs such as TRAF6 and IRAK1 could result in robust suppression of the innate immune response by blocking both the MYD88 as well as TRIF signaling pathways. In our earlier report, we showed that αA crystallin was upregulated in early EAU, specifically in the photoreceptors of the retina (6, 7) Intravenous administration of αA crystallin downregulated genes involved in the immune response such as TLR signaling as well as Th1, Th2 and Th17 cytokines. Similarly in the present study, systemic administration of microRNA146a, revealed suppression of TLRs and their signaling molecules IRAK1 and TRAF6. Interestingly previously validated *in-vitro* studies showed that microRNA146a targets IRAK1 and TRAF6 (59–61). To determine biological functions of microRNA 146a, several techniques have been employed. One common approach used *in vitro* or *in vivo* was with either miRNA mimics (for overexpression) or complementary inhibitors (to block miRNA function). Therefore, in the current study we evaluated the effects of systemic administration of microRNA146a and 155 by using microRNA mimics of them on the inhibition of the EAU. Moreover, we used the qPCR array to detect the mRNA levels of TLRs and their signaling molecules and the Th1/Th2/Th17 cytokines by Multi-Analyte ELISArray as we reported earlier (7). The innate as well as adaptive immune response genes were suppressed with miRNA 146a mimic administration in the retina as well as in the spleen (**Figure 5** and **Table 3**). We also determined the levels of the T reg activation marker FOXP3 in the spleen since T regs cells are known to suppress EAU (51). The microRNA 146a treatment resulted in upregulation of T regs. The combination of suppression of innate, adaptive immune response genes and upregulation of T reg from the microRNA 146a administration could have abrogated EAU. microRNA146a was also reported to suppress exacerbation of inflammation in allergic conjunctivitis by upregulation of FOXP3 and downregulation of pSTAT3 thus demonstrating a protective mechanism (62). Transgenic mice with altered miRNA expression have also been used successfully to study miRNA function, and have been designed to target miRNA expression at certain stages during development, as well as in a tissue- or cell type- specific manner (63, 64). Therefore in our study we showed the protective function of microRNA 146a in EAU, using microRNA146a KO mice. Interestingly our results revealed that the microRNA 146a KO mice had severe inflammation compared to the WT mice **(** **Figures 2**, **3****)** which substantiated our findings that microRNA146a can suppress uveitis and absence of this microRNA can lead to severe intraocular inflammation and retinal damage **(** **Figures 4**, **5****).** As local antigen presentation in the retina is critical for uveitis development, we designed a study to investigate the protective role of microRNA146a through local delivery and to see if such delivery would inhibit the TLRs signals in the retina resulting in inhibition of EAU. Our data indicated that similar to the protective effects of systemic administration of microRNA146a, the intravitreal local delivery of this microRNA also prevented development of uveitis. This finding indicated that the protection from local delivery could be from suppression of TLR signals required for local antigen presentation in the retina. To confirm the entry of the microRNA into the retina of these mice, localization of the microRNA after intravitreal delivery was carried out by confocal microscopy by using microRNA mimics labeled with a fluorescent dye DY547 as described by others (65). This enabled us to trace the injected microRNA and localize it in the retina **(** **Figure 6****).** Finally, to investigate the molecular mechanism of amelioration of uveitis by intravitreal local delivery of microRNA146a, we designed an experiment to study the immune response genes by qPCR array. We analyzed the TLRs and its signaling molecules as well as the Th1/Th2/Th17 cytokines in the spleen and the retina after intravitreal local delivery of microRNA146a into the eyes of EAU mice and compared them to the scrambled RNA delivered EAU eyes. In the spleen the TLRs signaling molecules IRAK1 and TRAF6 were upregulated in both microRNA146a, and scrambled RNA treated mice whereas in the retina these molecules were downregulated with microRNA 146a delivery and not with scrambled RNA **(** **Figure 8****).** The results indicate that the most likely mechanism of abrogation of uveitis with local delivery of microRNA146a could be due to suppression of local innate immune response that could translate into suppression of Th1/Th2/Th17 immune response in EAU. These data suggests that intravitreal delivery of microRNA146a may abrogate EAU, however additional experimental evidence is required to determine the mechanism required for such abrogation of EAU inflammatory process. Overexpression of microRNA146a also showed a similar result by demonstrating decreased expression of proinflammatory mediators TRAF6 and IRAK1 and downstream target NFkB and other inflammatory cytokines such as IL-1α, IL-1 *β* and IL-6 in diabetic cornea (66). A similar study in EAAU in rats following miR-146a injections downregulation of interleukin- (IL-) 1*β*, IL-6, IL-12 and IFN *γ* and upregulation of IL-10 and IL-17 were noted (56). MicroRNAs are known to be transcribed in the nucleus by the transcription factor NFkB (45) and are exported to the cytoplasm where they control the expression of various target mRNAs. Through this mechanism they serve as a new class of novel target specific immune suppressors and can be used as a novel therapeutic agent in autoimmune diseases (54). In our study, administration of αA crystallin in EAU upregulated the microRNA146a, therefore indicating a possible mechanism of interaction of αA crystallin with NFκB. Since microRNA biogenesis occurs through activation of NFκB, in our EAU model αA crystallin may activate this transcription factor. NFκB activation can occur through the TLR signaling pathway by the activation of its signaling molecules IRAK1 and TRAF6. In addition to TLR signaling, TNF α is also known to activate NFκB. However, till date the role of αA crystallin in activation of NFκB is unknown. A recent report with Hsp27 a close member of crystallin family had demonstrated that Hsp27 regulates NFkB activity through the degradation of iκB (18). Similar to this observation, αA crystallin might also activate NFκB through iκB degradation. To investigate this function of αA crystallin, we further tested the activation of NFκB by αA crystallin administration in αA KO mice with EAU. αA KO mice proved to be a good model for this study since there is no endogenous αA crystallin and the molecular action we detect is solely due to the exogenous αA crystallin administration through intravenous injection. The results showed that, *in vivo* administration of αA crystallin in αA KO mice resulted in NFκBp65 activation. There was increased phosphorylation of iκB and activated NFκBp65 in these mice administered with αA crystallin compared to the control animals **(** **Figure 1****)**, and such activation of NFκB by αA crystallin is known to lead to the upregulation of microRNA146a as reported by others (54). However further studies are required to confirm its role in our experimental conditions. In depth studies in the IRBP-induced EAU model will be done to investigate whether NF-kB pathway plays a role in this model and if it does, how the administration of α a crystallin affect its pathway.

Current treatments for uveitis are associated with various side effects because of their broader immune suppression and the targeting of immune cells or cytokines. Therefore, microRNAs as modulators of cytokine genes offer an innovative approach to the treatment of uveitis. Such studies on microRNAs also have a broader implication in the treatment of various ocular, neurologic and other organ specific inflammatory diseases. Identifying the targets of miRNAs in inflammation, which have multicellular components is a great challenge, and one of the most important goals to advance our understanding of how miRNAs contribute to health and disease. Continued research into the regulatory powers of these small non-coding RNAs is required to fully determine their potential for the treatment of disease which will enable them to be successfully targeted and delivered to patients and bring new therapies to the clinic.

## Data availability statement

The original contributions presented in the study are included in the article/supplementary material. Further inquiries can be directed to the corresponding author.

## Ethics statement

The animal study was reviewed and approved by Guide for the Care and Use of Laboratory Animals of the Association of Research in Vision and Ophthalmology. The protocol was approved by the Committee of the Ethics of Animal Experiments of the University of Southern California (Protocol Number: 11218).

## Author contributions

SS: Designed and Performed all experiments. Contributed in analyzing the data, data compilations, preparing images for figures and writing the manuscript. NR: Guidance throughout the project in designing the experiments and reviewing the results, and analyzing data and review the manuscript. All authors contributed to the article and approved the submitted version.
